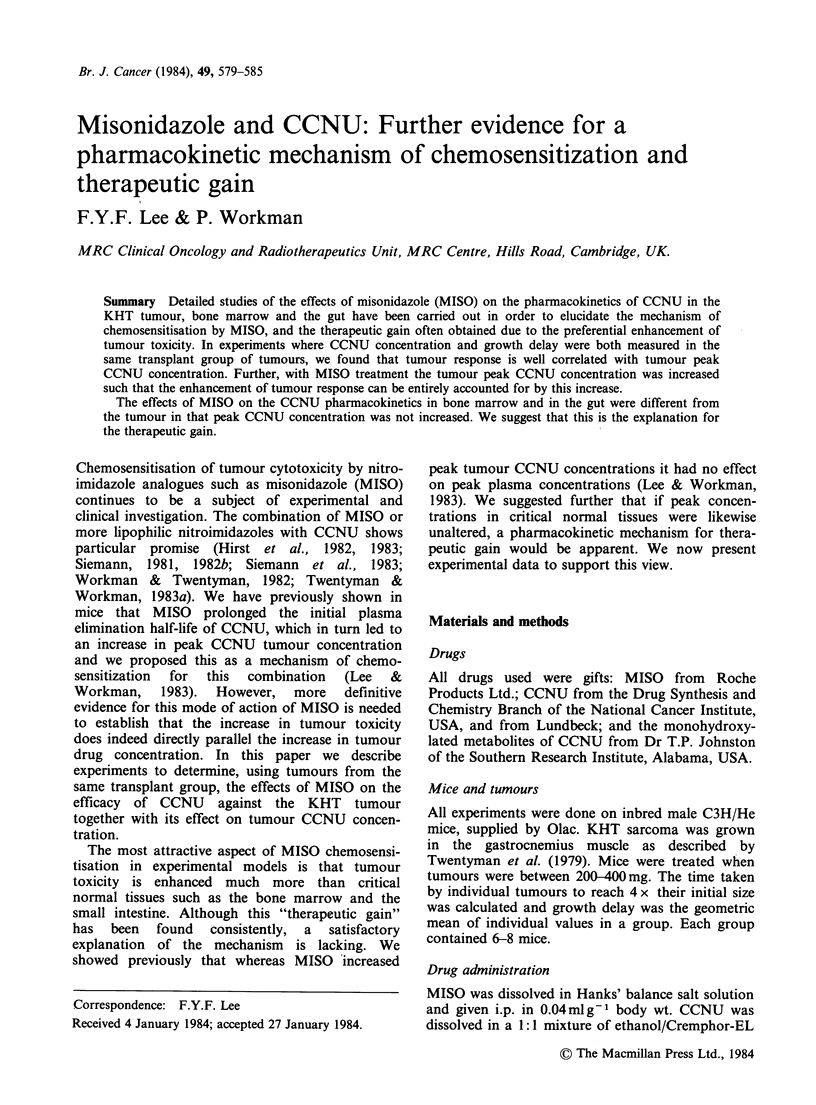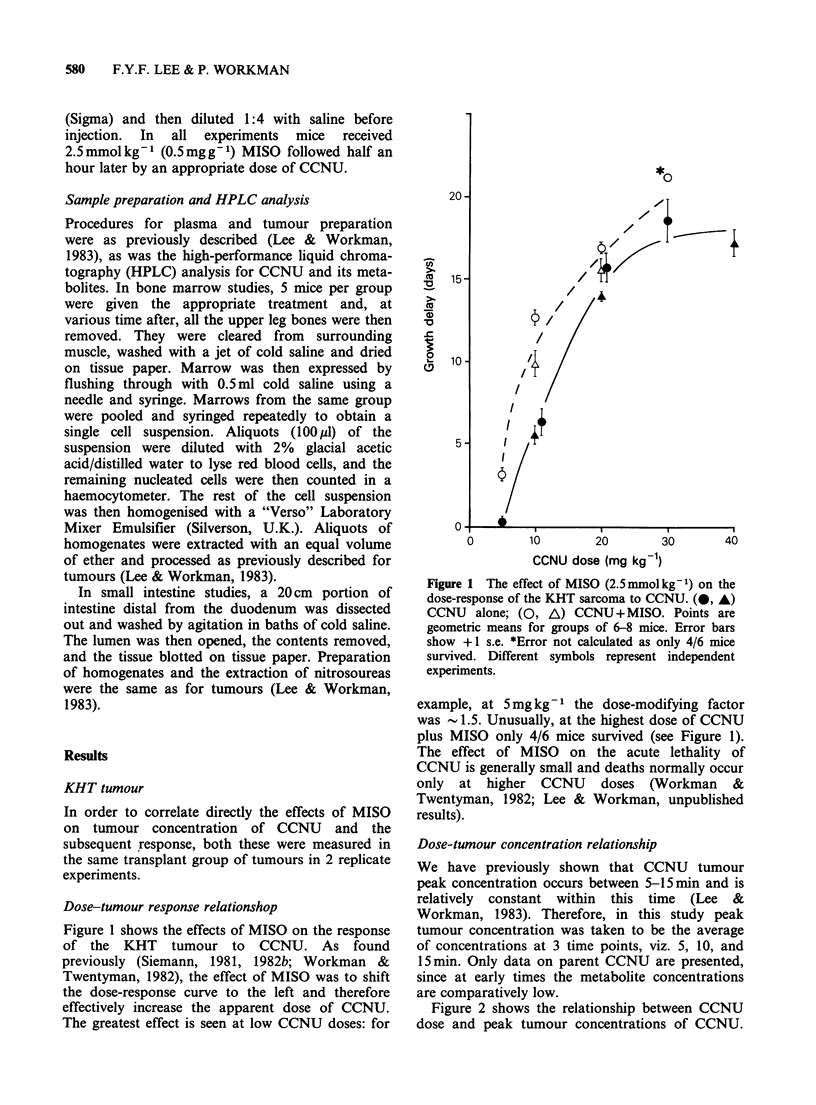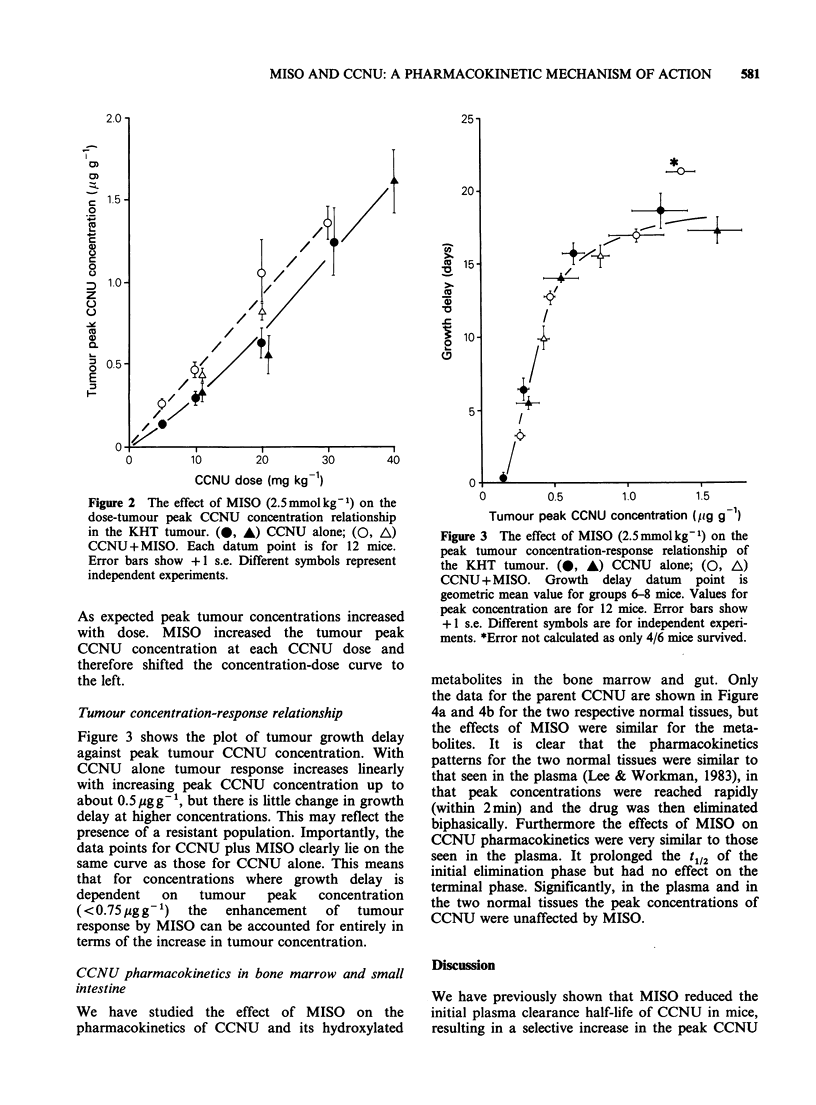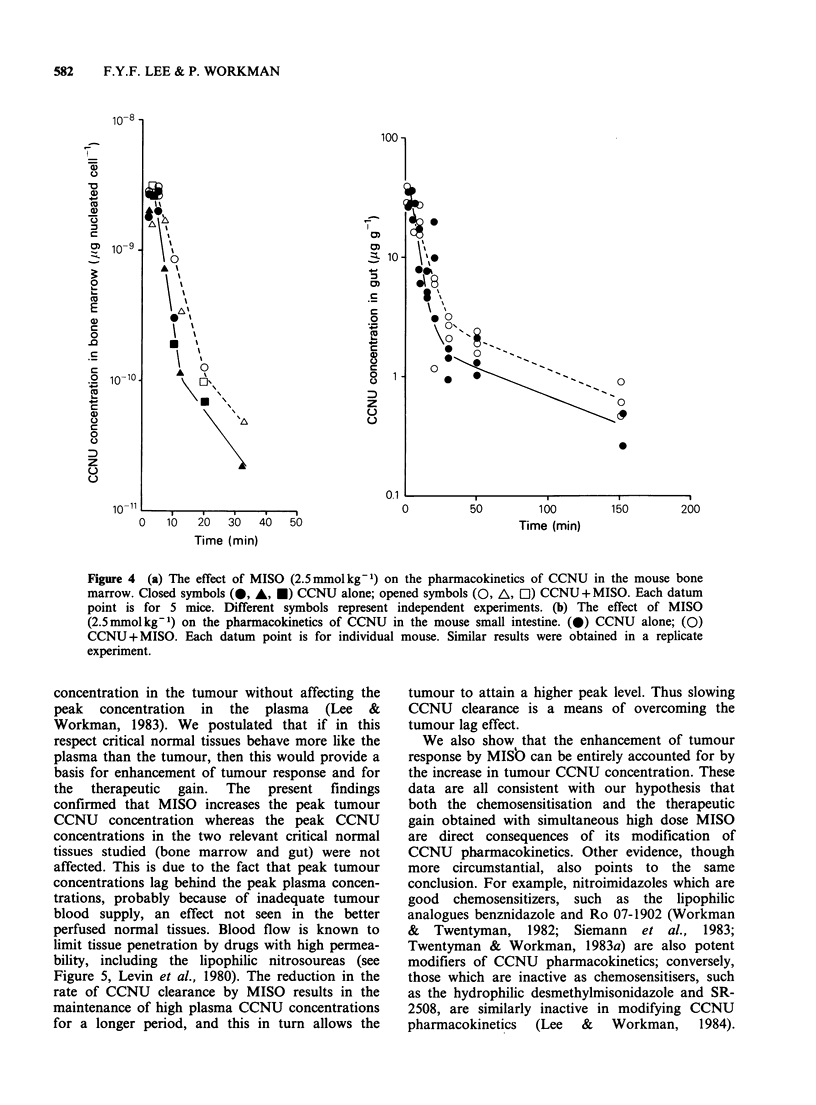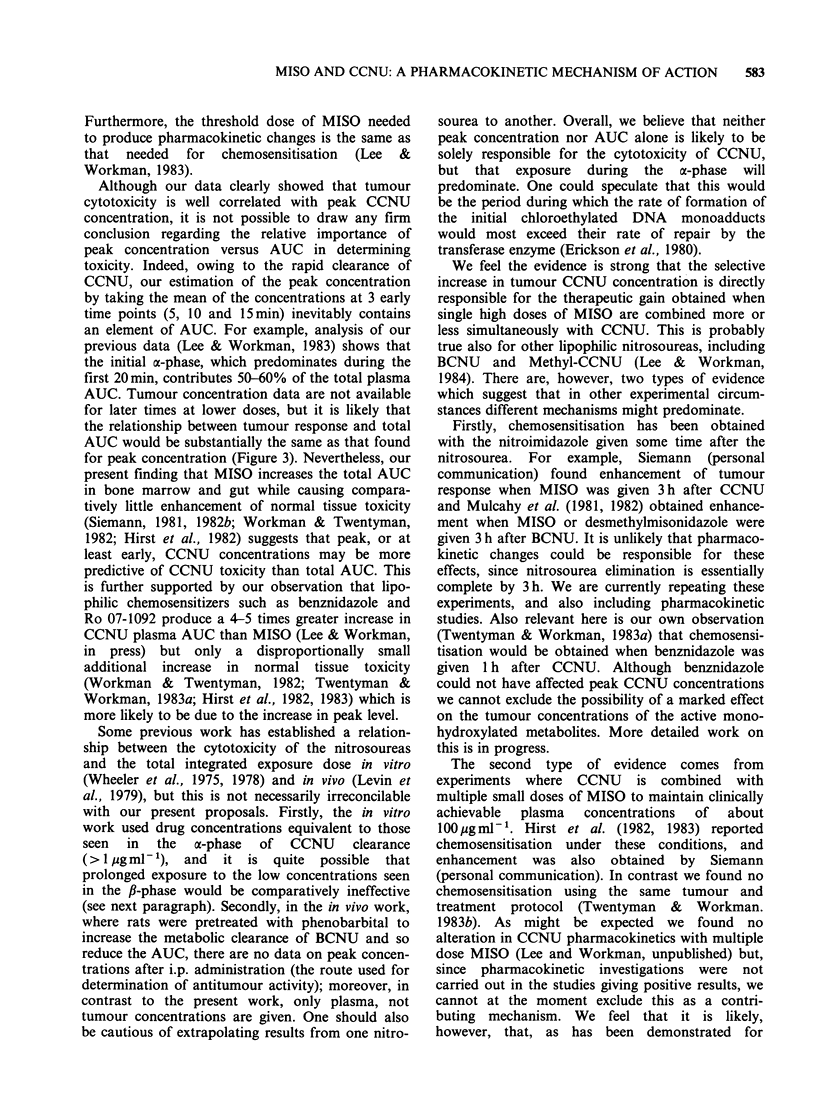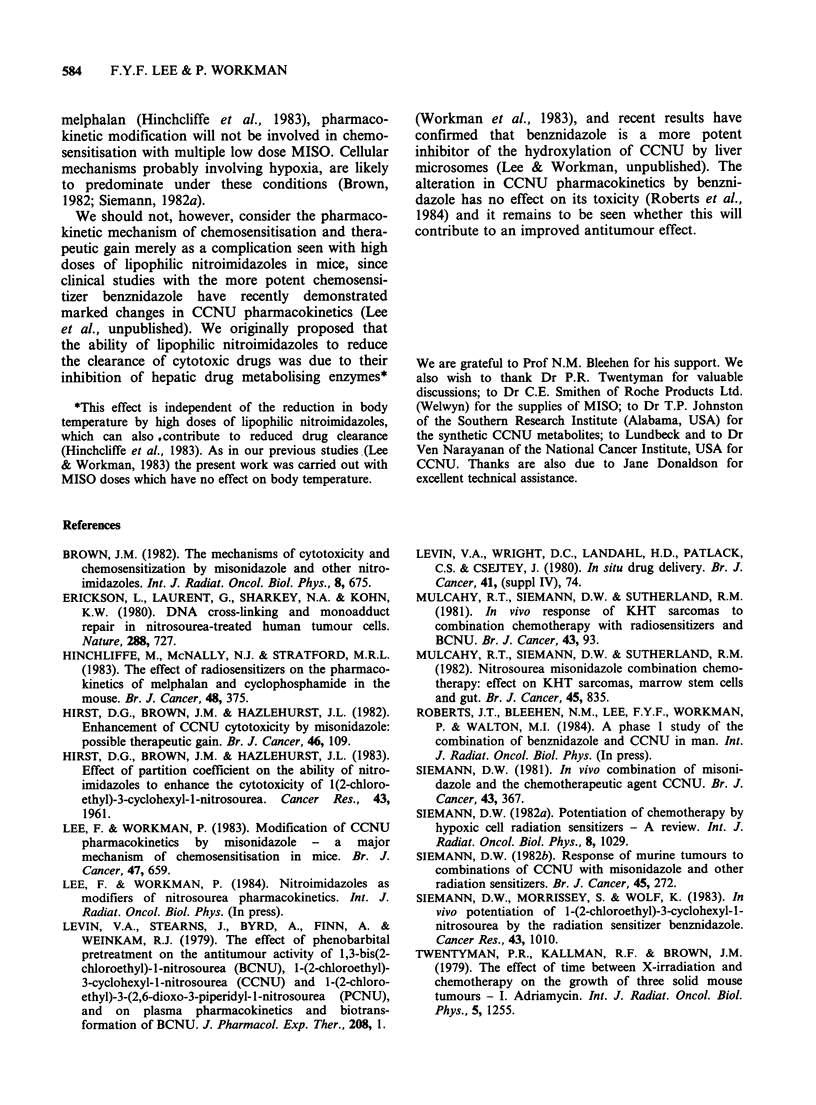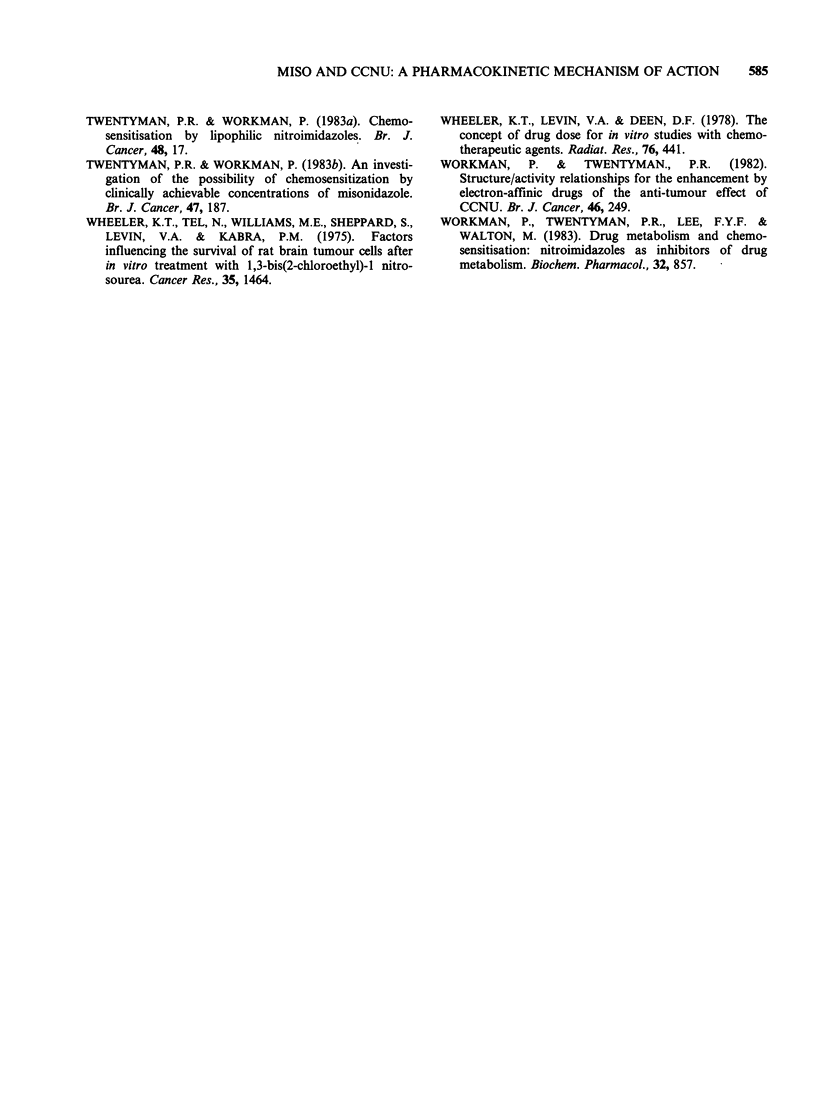# Misonidazole and CCNU: further evidence for a pharmacokinetic mechanism of chemosensitization and therapeutic gain.

**DOI:** 10.1038/bjc.1984.92

**Published:** 1984-05

**Authors:** F. Y. Lee, P. Workman

## Abstract

Detailed studies of the effects of misonidazole (MISO) on the pharmacokinetics of CCNU in the KHT tumour, bone marrow and the gut have been carried out in order to elucidate the mechanism of chemosensitisation by MISO, and the therapeutic gain often obtained due to the preferential enhancement of tumour toxicity. In experiments where CCNU concentration and growth delay were both measured in the same transplant group of tumours, we found that tumour response is well correlated with tumour peak CCNU concentration. Further, with MISO treatment the tumour peak CCNU concentration was increased such that the enhancement of tumour response can be entirely accounted for by this increase. The effects of MISO on the CCNU pharmacokinetics in bone marrow and in the gut were different from the tumour in that peak CCNU concentration was not increased. We suggest that this is the explanation for the therapeutic gain.


Br. J. Cancer (1984), **49**, 579–585

### Misonidazole and CCNU: Further evidence for a pharmacokinetic mechanism of chemosensitization and therapeutic gain

F.Y.F. Lee & P. Workman

*MRC Clinical Oncology and Radiotherapeutics Unit, MRC Centre, Hills Road, Cambridge, UK.*

**Summary** Detailed studies of the effects of misonidazole (MISO) on the pharmacokinetics of CCNU in the KHT tumour, bone marrow and the gut have been carried out in order to elucidate the mechanism of chemosensitisation by MISO, and the therapeutic gain often obtained due to the preferential enhancement of tumour toxicity. In experiments where CCNU concentration and growth delay were both measured in the same transplant group of tumours, we found that tumour response is well correlated with tumour peak CCNU concentration. Further, with MISO treatment the tumour peak CCNU concentration was increased such that the enhancement of tumour response can be entirely accounted for by this increase.

The effects of MISO on the CCNU pharmacokinetics in bone marrow and in the gut were different from the tumour in that peak CCNU concentration was not increased. We suggest that this is the explanation for the therapeutic gain.

Chemosensitisation of tumour cytotoxicity by nitroimidazole analogues such as misonidazole (MISO) continues to be a subject of experimental and clinical investigation. The combination of MISO or more lipophilic nitroimidazoles with CCNU shows particular promise (Hirst *et al.*, 1982, 1983; Siemann, 1981, 1982*b*; Siemann *et al.*, 1983; Workman & Twentyman, 1982; Twentyman & Workman, 1983*a*). We have previously shown in mice that MISO prolonged the initial plasma elimination half-life of CCNU, which in turn led to an increase in peak CCNU tumour concentration and we proposed this as a mechanism of chemosensitization for this combination (Lee & Workman, 1983). However, more definitive evidence for this mode of action of MISO is needed to establish that the increase in tumour toxicity does indeed directly parallel the increase in tumour drug concentration. In this paper we describe experiments to determine, using tumours from the same transplant group, the effects of MISO on the efficacy of CCNU against the KHT tumour together with its effect on tumour CCNU concentration.

The most attractive aspect of MISO chemosensitisation in experimental models is that tumour toxicity is enhanced much more than critical normal tissues such as the bone marrow and the small intestine. Although this "therapeutic gain" has been found consistently, a satisfactory explanation of the mechanism is lacking. We showed previously that whereas MISO increased peak tumour CCNU concentrations it had no effect on peak plasma concentrations (Lee & Workman, 1983). We suggested further that if peak concentrations in critical normal tissues were likewise unaltered, a pharmacokinetic mechanism for therapeutic gain would be apparent. We now present experimental data to support this view.

Correspondence: F.Y.F. Lee

Received 4 January 1984; accepted 27 January 1984.

### Materials and methods

### *Drugs*

All drugs used were gifts: MISO from Roche Products Ltd.; CCNU from the Drug Synthesis and Chemistry Branch of the National Cancer Institute, USA, and from Lundbeck; and the monohydroxylated metabolites of CCNU from Dr T.P. Johnston of the Southern Research Institute, Alabama, USA.

### *Mice and tumours*

All experiments were done on inbred male C3H/He mice, supplied by Olac. KHT sarcoma was grown in the gastrocnemius muscle as described by Twentyman *et al.* (1979). Mice were treated when tumours were between 200–400 mg. The time taken by individual tumours to reach 4 × their initial size was calculated and growth delay was the geometric mean of individual values in a group. Each group contained 6–8 mice.

### *Drug administration*

MISO was dissolved in Hanks' balance salt solution and given i.p. in 0.04 ml $g^{-1}$ body wt. CCNU was dissolved in a 1:1 mixture of ethanol/Cremphor-EL

© The Macmillan Press Ltd., 1984

(Sigma) and then diluted 1:4 with saline before injection. In all experiments mice received 2.5 mmol $kg^{-1}$ (0.5 mg $g^{-1}$) MISO followed half an hour later by an appropriate dose of CCNU.

*Sample preparation and HPLC analysis*

Procedures for plasma and tumour preparation were as previously described (Lee & Workman, 1983), as was the high-performance liquid chromatography (HPLC) analysis for CCNU and its metabolites. In bone marrow studies, 5 mice per group were given the appropriate treatment and, at various time after, all the upper leg bones were then removed. They were cleared from surrounding muscle, washed with a jet of cold saline and dried on tissue paper. Marrow was then expressed by flushing through with 0.5 ml cold saline using a needle and syringe. Marrows from the same group were pooled and syringed repeatedly to obtain a single cell suspension. Aliquots (100 µl) of the suspension were diluted with 2% glacial acetic acid/distilled water to lyse red blood cells, and the remaining nucleated cells were then counted in a haemocytometer. The rest of the cell suspension was then homogenised with a "Verso" Laboratory Mixer Emulsifier (Silverson, U.K.). Aliquots of homogenates were extracted with an equal volume of ether and processed as previously described for tumours (Lee & Workman, 1983).

In small intestine studies, a 20 cm portion of intestine distal from the duodenum was dissected out and washed by agitation in baths of cold saline. The lumen was then opened, the contents removed, and the tissue blotted on tissue paper. Preparation of homogenates and the extraction of nitrosoureas were the same as for tumours (Lee & Workman, 1983).

### Results

*KHT tumour*

In order to correlate directly the effects of MISO on tumour concentration of CCNU and the subsequent response, both these were measured in the same transplant group of tumours in 2 replicate experiments.

*Dose–tumour response relationshop*

Figure 1 shows the effects of MISO on the response of the KHT tumour to CCNU. As found previously (Siemann, 1981, 1982*b*; Workman & Twentyman, 1982), the effect of MISO was to shift the dose-response curve to the left and therefore effectively increase the apparent dose of CCNU. The greatest effect is seen at low CCNU doses: for

**Figure 1** The effect of MISO (2.5 mmol $kg^{-1}$) on the dose-response of the KHT sarcoma to CCNU. (●, ▲) CCNU alone; (○, △) CCNU+MISO. Points are geometric means for groups of 6–8 mice. Error bars show +1 s.e. *Error not calculated as only 4/6 mice survived. Different symbols represent independent experiments.

example, at 5 mg $kg^{-1}$ the dose-modifying factor was ~1.5. Unusually, at the highest dose of CCNU plus MISO only 4/6 mice survived (see Figure 1). The effect of MISO on the acute lethality of CCNU is generally small and deaths normally occur only at higher CCNU doses (Workman & Twentyman, 1982; Lee & Workman, unpublished results).

*Dose-tumour concentration relationship*

We have previously shown that CCNU tumour peak concentration occurs between 5–15 min and is relatively constant within this time (Lee & Workman, 1983). Therefore, in this study peak tumour concentration was taken to be the average of concentrations at 3 time points, viz. 5, 10, and 15 min. Only data on parent CCNU are presented, since at early times the metabolite concentrations are comparatively low.

Figure 2 shows the relationship between CCNU dose and peak tumour concentrations of CCNU.

**Figure 2** The effect of MISO (2.5 mmol kg$^{-1}$) on the dose-tumour peak CCNU concentration relationship in the KHT tumour. (●, ▲) CCNU alone; (○, △) CCNU+MISO. Each datum point is for 12 mice. Error bars show +1 s.e. Different symbols represent independent experiments.

**Figure 3** The effect of MISO (2.5 mmol kg$^{-1}$) on the peak tumour concentration-response relationship of the KHT tumour. (●, ▲) CCNU alone; (○, △) CCNU+MISO. Growth delay datum point is geometric mean value for groups 6–8 mice. Values for peak concentration are for 12 mice. Error bars show +1 s.e. Different symbols are for independent experiments. *Error not calculated as only 4/6 mice survived.

As expected peak tumour concentrations increased with dose. MISO increased the tumour peak CCNU concentration at each CCNU dose and therefore shifted the concentration-dose curve to the left.

*Tumour concentration-response relationship*

Figure 3 shows the plot of tumour growth delay against peak tumour CCNU concentration. With CCNU alone tumour response increases linearly with increasing peak CCNU concentration up to about 0.5 $\mu$g g$^{-1}$, but there is little change in growth delay at higher concentrations. This may reflect the presence of a resistant population. Importantly, the data points for CCNU plus MISO clearly lie on the same curve as those for CCNU alone. This means that for concentrations where growth delay is dependent on tumour peak concentration ($<0.75\,\mu$g g$^{-1}$) the enhancement of tumour response by MISO can be accounted for entirely in terms of the increase in tumour concentration.

*CCNU pharmacokinetics in bone marrow and small intestine*

We have studied the effect of MISO on the pharmacokinetics of CCNU and its hydroxylated metabolites in the bone marrow and gut. Only the data for the parent CCNU are shown in Figure 4a and 4b for the two respective normal tissues, but the effects of MISO were similar for the metabolites. It is clear that the pharmacokinetics patterns for the two normal tissues were similar to that seen in the plasma (Lee & Workman, 1983), in that peak concentrations were reached rapidly (within 2 min) and the drug was then eliminated biphasically. Furthermore the effects of MISO on CCNU pharmacokinetics were very similar to those seen in the plasma. It prolonged the $t_{1/2}$ of the initial elimination phase but had no effect on the terminal phase. Significantly, in the plasma and in the two normal tissues the peak concentrations of CCNU were unaffected by MISO.

### Discussion

We have previously shown that MISO reduced the initial plasma clearance half-life of CCNU in mice, resulting in a selective increase in the peak CCNU

**Figure 4** (**a**) The effect of MISO (2.5 mmol kg$^{-1}$) on the pharmacokinetics of CCNU in the mouse bone marrow. Closed symbols (●, ▲, ■) CCNU alone; opened symbols (○, △, □) CCNU + MISO. Each datum point is for 5 mice. Different symbols represent independent experiments. (**b**) The effect of MISO (2.5 mmol kg$^{-1}$) on the pharmacokinetics of CCNU in the mouse small intestine. (●) CCNU alone; (○) CCNU + MISO. Each datum point is for individual mouse. Similar results were obtained in a replicate experiment.

concentration in the tumour without affecting the peak concentration in the plasma (Lee & Workman, 1983). We postulated that if in this respect critical normal tissues behave more like the plasma than the tumour, then this would provide a basis for enhancement of tumour response and for the therapeutic gain. The present findings confirmed that MISO increases the peak tumour CCNU concentration whereas the peak CCNU concentrations in the two relevant critical normal tissues studied (bone marrow and gut) were not affected. This is due to the fact that peak tumour concentrations lag behind the peak plasma concentrations, probably because of inadequate tumour blood supply, an effect not seen in the better perfused normal tissues. Blood flow is known to limit tissue penetration by drugs with high permeability, including the lipophilic nitrosoureas (see Figure 5, Levin *et al.*, 1980). The reduction in the rate of CCNU clearance by MISO results in the maintenance of high plasma CCNU concentrations for a longer period, and this in turn allows the tumour to attain a higher peak level. Thus slowing CCNU clearance is a means of overcoming the tumour lag effect.

We also show that the enhancement of tumour response by MISO can be entirely accounted for by the increase in tumour CCNU concentration. These data are all consistent with our hypothesis that both the chemosensitisation and the therapeutic gain obtained with simultaneous high dose MISO are direct consequences of its modification of CCNU pharmacokinetics. Other evidence, though more circumstantial, also points to the same conclusion. For example, nitroimidazoles which are good chemosensitizers, such as the lipophilic analogues benznidazole and Ro 07-1902 (Workman & Twentyman, 1982; Siemann *et al.*, 1983; Twentyman & Workman, 1983*a*) are also potent modifiers of CCNU pharmacokinetics; conversely, those which are inactive as chemosensitisers, such as the hydrophilic desmethylmisonidazole and SR-2508, are similarly inactive in modifying CCNU pharmacokinetics (Lee & Workman, 1984).

Furthermore, the threshold dose of MISO needed to produce pharmacokinetic changes is the same as that needed for chemosensitisation (Lee & Workman, 1983).

Although our data clearly showed that tumour cytotoxicity is well correlated with peak CCNU concentration, it is not possible to draw any firm conclusion regarding the relative importance of peak concentration versus AUC in determining toxicity. Indeed, owing to the rapid clearance of CCNU, our estimation of the peak concentration by taking the mean of the concentrations at 3 early time points (5, 10 and 15 min) inevitably contains an element of AUC. For example, analysis of our previous data (Lee & Workman, 1983) shows that the initial α-phase, which predominates during the first 20 min, contributes 50–60% of the total plasma AUC. Tumour concentration data are not available for later times at lower doses, but it is likely that the relationship between tumour response and total AUC would be substantially the same as that found for peak concentration (Figure 3). Nevertheless, our present finding that MISO increases the total AUC in bone marrow and gut while causing comparatively little enhancement of normal tissue toxicity (Siemann, 1981, 1982*b*; Workman & Twentyman, 1982; Hirst *et al.*, 1982) suggests that peak, or at least early, CCNU concentrations may be more predictive of CCNU toxicity than total AUC. This is further supported by our observation that lipophilic chemosensitizers such as benznidazole and Ro 07-1092 produce a 4–5 times greater increase in CCNU plasma AUC than MISO (Lee & Workman, in press) but only a disproportionally small additional increase in normal tissue toxicity (Workman & Twentyman, 1982; Twentyman & Workman, 1983*a*; Hirst *et al.*, 1982, 1983) which is more likely to be due to the increase in peak level.

Some previous work has established a relationship between the cytotoxicity of the nitrosoureas and the total integrated exposure dose *in vitro* (Wheeler *et al.*, 1975, 1978) and *in vivo* (Levin *et al.*, 1979), but this is not necessarily irreconcilable with our present proposals. Firstly, the *in vitro* work used drug concentrations equivalent to those seen in the α-phase of CCNU clearance ($> 1\,\mu g\,ml^{-1}$), and it is quite possible that prolonged exposure to the low concentrations seen in the β-phase would be comparatively ineffective (see next paragraph). Secondly, in the *in vivo* work, where rats were pretreated with phenobarbital to increase the metabolic clearance of BCNU and so reduce the AUC, there are no data on peak concentrations after i.p. administration (the route used for determination of antitumour activity); moreover, in contrast to the present work, only plasma, not tumour concentrations are given. One should also be cautious of extrapolating results from one nitrosourea to another. Overall, we believe that neither peak concentration nor AUC alone is likely to be solely responsible for the cytotoxicity of CCNU, but that exposure during the α-phase will predominate. One could speculate that this would be the period during which the rate of formation of the initial chloroethylated DNA monoadducts would most exceed their rate of repair by the transferase enzyme (Erickson *et al.*, 1980).

We feel the evidence is strong that the selective increase in tumour CCNU concentration is directly responsible for the therapeutic gain obtained when single high doses of MISO are combined more or less simultaneously with CCNU. This is probably true also for other lipophilic nitrosoureas, including BCNU and Methyl-CCNU (Lee & Workman, 1984). There are, however, two types of evidence which suggest that in other experimental circumstances different mechanisms might predominate.

Firstly, chemosensitisation has been obtained with the nitroimidazole given some time after the nitrosourea. For example, Siemann (personal communication) found enhancement of tumour response when MISO was given 3 h after CCNU and Mulcahy *et al.* (1981, 1982) obtained enhancement when MISO or desmethylmisonidazole were given 3 h after BCNU. It is unlikely that pharmacokinetic changes could be responsible for these effects, since nitrosourea elimination is essentially complete by 3 h. We are currently repeating these experiments, and also including pharmacokinetic studies. Also relevant here is our own observation (Twentyman & Workman, 1983*a*) that chemosensitisation would be obtained when benznidazole was given 1 h after CCNU. Although benznidazole could not have affected peak CCNU concentrations we cannot exclude the possibility of a marked effect on the tumour concentrations of the active monohydroxylated metabolites. More detailed work on this is in progress.

The second type of evidence comes from experiments where CCNU is combined with multiple small doses of MISO to maintain clinically achievable plasma concentrations of about $100\,\mu g\,ml^{-1}$. Hirst *et al.* (1982, 1983) reported chemosensitisation under these conditions, and enhancement was also obtained by Siemann (personal communication). In contrast we found no chemosensitisation using the same tumour and treatment protocol (Twentyman & Workman. 1983*b*). As might be expected we found no alteration in CCNU pharmacokinetics with multiple dose MISO (Lee and Workman, unpublished) but, since pharmacokinetic investigations were not carried out in the studies giving positive results, we cannot at the moment exclude this as a contributing mechanism. We feel that it is likely, however, that, as has been demonstrated for melphalan (Hinchcliffe *et al.*, 1983), pharmacokinetic modification will not be involved in chemosensitisation with multiple low dose MISO. Cellular mechanisms probably involving hypoxia, are likely to predominate under these conditions (Brown, 1982; Siemann, 1982*a*).

We should not, however, consider the pharmacokinetic mechanism of chemosensitisation and therapeutic gain merely as a complication seen with high doses of lipophilic nitroimidazoles in mice, since clinical studies with the more potent chemosensitizer benznidazole have recently demonstrated marked changes in CCNU pharmacokinetics (Lee *et al.*, unpublished). We originally proposed that the ability of lipophilic nitroimidazoles to reduce the clearance of cytotoxic drugs was due to their inhibition of hepatic drug metabolising enzymes* (Workman *et al.*, 1983), and recent results have confirmed that benznidazole is a more potent inhibitor of the hydroxylation of CCNU by liver microsomes (Lee & Workman, unpublished). The alteration in CCNU pharmacokinetics by benznidazole has no effect on its toxicity (Roberts *et al.*, 1984) and it remains to be seen whether this will contribute to an improved antitumour effect.

*This effect is independent of the reduction in body temperature by high doses of lipophilic nitroimidazoles, which can also contribute to reduced drug clearance (Hinchcliffe *et al.*, 1983). As in our previous studies (Lee & Workman, 1983) the present work was carried out with MISO doses which have no effect on body temperature.

We are grateful to Prof N.M. Bleehen for his support. We also wish to thank Dr P.R. Twentyman for valuable discussions; to Dr C.E. Smithen of Roche Products Ltd. (Welwyn) for the supplies of MISO; to Dr T.P. Johnston of the Southern Research Institute (Alabama, USA) for the synthetic CCNU metabolites; to Lundbeck and to Dr Ven Narayanan of the National Cancer Institute, USA for CCNU. Thanks are also due to Jane Donaldson for excellent technical assistance.

### References

BROWN, J.M. (1982). The mechanisms of cytotoxicity and chemosensitization by misonidazole and other nitroimidazoles. *Int. J. Radiat. Oncol. Biol. Phys.*, **8**, 675.

ERICKSON, L., LAURENT, G., SHARKEY, N.A. & KOHN, K.W. (1980). DNA cross-linking and monoadduct repair in nitrosourea-treated human tumour cells. *Nature*, **288**, 727.

HINCHLIFFE, M., McNALLY, N.J. & STRATFORD, M.R.L. (1983). The effect of radiosensitizers on the pharmacokinetics of melphalan and cyclophosphamide in the mouse. *Br. J. Cancer*, **48**, 375.

HIRST, D.G., BROWN, J.M. & HAZLEHURST, J.L. (1982). Enhancement of CCNU cytotoxicity by misonidazole: possible therapeutic gain. *Br. J. Cancer*, **46**, 109.

HIRST, D.G., BROWN, J.M. & HAZLEHURST, J.L. (1983). Effect of partition coefficient on the ability of nitroimidazoles to enhance the cytotoxicity of 1(2-chloroethyl)-3-cyclohexyl-1-nitrosourea. *Cancer Res.*, **43**, 1961.

LEE, F. & WORKMAN, P. (1983). Modification of CCNU pharmacokinetics by misonidazole – a major mechanism of chemosensitisation in mice. *Br. J. Cancer*, **47**, 659.

LEE, F. & WORKMAN, P. (1984). Nitroimidazoles as modifiers of nitrosourea pharmacokinetics. *Int. J. Radiat. Oncol. Biol. Phys.* (In press).

LEVIN, V.A., STEARNS, J., BYRD, A., FINN, A. & WEINKAM, R.J. (1979). The effect of phenobarbital pretreatment on the antitumour activity of 1,3-bis(2-chloroethyl)-1-nitrosourea (BCNU), 1-(2-chloroethyl)-3-cyclohexyl-1-nitrosourea (CCNU) and 1-(2-chloroethyl)-3-(2,6-dioxo-3-piperidyl-1-nitrosourea (PCNU), and on plasma pharmacokinetics and biotransformation of BCNU. *J. Pharmacol. Exp. Ther.*, **208**, 1.

LEVIN, V.A., WRIGHT, D.C., LANDAHL, H.D., PATLACK, C.S. & CSEJTEY, J. (1980). *In situ* drug delivery. *Br. J. Cancer*, **41**, (suppl IV), 74.

MULCAHY, R.T., SIEMANN, D.W. & SUTHERLAND, R.M. (1981). *In vivo* response of KHT sarcomas to combination chemotherapy with radiosensitizers and BCNU. *Br. J. Cancer*, **43**, 93.

MULCAHY, R.T., SIEMANN, D.W. & SUTHERLAND, R.M. (1982). Nitrosourea misonidazole combination chemotherapy: effect on KHT sarcomas, marrow stem cells and gut. *Br. J. Cancer*, **45**, 835.

ROBERTS, J.T., BLEEHEN, N.M., LEE, F.Y.F., WORKMAN, P. & WALTON, M.I. (1984). A phase 1 study of the combination of benznidazole and CCNU in man. *Int. J. Radiat. Oncol. Biol. Phys.* (In press).

SIEMANN, D.W. (1981). *In vivo* combination of misonidazole and the chemotherapeutic agent CCNU. *Br. J. Cancer*, **43**, 367.

SIEMANN, D.W. (1982*a*). Potentiation of chemotherapy by hypoxic cell radiation sensitizers – A review. *Int. J. Radiat. Oncol. Biol. Phys.*, **8**, 1029.

SIEMANN, D.W. (1982*b*). Response of murine tumours to combinations of CCNU with misonidazole and other radiation sensitizers. *Br. J. Cancer*, **45**, 272.

SIEMANN, D.W., MORRISSEY, S. & WOLF, K. (1983). *In vivo* potentiation of 1-(2-chloroethyl)-3-cyclohexyl-1-nitrosourea by the radiation sensitizer benznidazole. *Cancer Res.*, **43**, 1010.

TWENTYMAN, P.R., KALLMAN, R.F. & BROWN, J.M. (1979). The effect of time between X-irradiation and chemotherapy on the growth of three solid mouse tumours – I. Adriamycin. *Int. J. Radiat. Oncol. Biol. Phys.*, **5**, 1255.

TWENTYMAN, P.R. & WORKMAN, P. (1983*a*). Chemosensitisation by lipophilic nitroimidazoles. *Br. J. Cancer,* **48,** 17.

TWENTYMAN, P.R. & WORKMAN, P. (1983*b*). An investigation of the possibility of chemosensitization by clinically achievable concentrations of misonidazole. *Br. J. Cancer,* **47,** 187.

WHEELER, K.T., TEL, N., WILLIAMS, M.E., SHEPPARD, S., LEVIN, V.A. & KABRA, P.M. (1975). Factors influencing the survival of rat brain tumour cells after *in vitro* treatment with 1,3-bis(2-chloroethyl)-1 nitrosourea. *Cancer Res.,* **35,** 1464.

WHEELER, K.T., LEVIN, V.A. & DEEN, D.F. (1978). The concept of drug dose for *in vitro* studies with chemotherapeutic agents. *Radiat. Res.,* **76,** 441.

WORKMAN, P. & TWENTYMAN., P.R. (1982). Structure/activity relationships for the enhancement by electron-affinic drugs of the anti-tumour effect of CCNU. *Br. J. Cancer,* **46,** 249.

WORKMAN, P., TWENTYMAN, P.R., LEE, F.Y.F. & WALTON, M. (1983). Drug metabolism and chemosensitisation: nitroimidazoles as inhibitors of drug metabolism. *Biochem. Pharmacol.,* **32,** 857.